# Coronary spasm as a manifestation of allergic reaction to contrast material: a case report of Kounis syndrome

**DOI:** 10.1093/ehjcr/ytaf473

**Published:** 2025-09-19

**Authors:** Ondrej Zmolik, Stephan Hainzl, Christoph Kohler, Stefan Sonnet, Marc Slawik

**Affiliations:** Department of Internal Medicine, St. Clara Hospital, Kleinriehenstrasse 30, Basel 4058, Switzerland; Department of Internal Medicine, St. Clara Hospital, Kleinriehenstrasse 30, Basel 4058, Switzerland; Department of Cardiology, St. Clara Hospital, Kleinriehenstrasse 30, Basel 4058, Switzerland; Department of Radiology, St. Clara Hospital, Kleinriehenstrasse 30, Basel 4058, Switzerland; Department of Internal Medicine, St. Clara Hospital, Kleinriehenstrasse 30, Basel 4058, Switzerland

**Keywords:** Kounis syndrome, Coronary vasospasm, Hypersensitivity reaction, Contrast media allergy, Case report, Acute coronary syndrome

## Abstract

**Background:**

Kounis syndrome is a rare but significant clinical entity that combines hypersensitivity reactions with acute coronary syndromes. It is triggered by various allergens, including contrast media, leading to coronary vasospasm, atheromatous plaque rupture, or stent thrombosis. Despite its clinical relevance, the diagnosis is often challenging due to its multifaceted presentation.

**Case summary:**

We report the case of a 60-year-old woman with a history of bronchial adenocarcinoma treated with pembrolizumab, who developed Kounis syndrome Type 1 following the administration of iodinated contrast media during a computed tomography scan. Shortly after receiving the contrast agent, the patient experienced acute thoracic pain, dyspnoea, and ST-segment elevations indicative of inferior ischaemia. Emergency coronary angiography revealed a coronary vasospasm without significant stenosis, which resolved upon administration of intracoronary nitroglycerin. The diagnosis of Kounis syndrome was further supported by delayed hypersensitivity symptoms, including a trunk-accentuated rash. Management included antihistamines, corticosteroids, and vasodilators, resulting in complete resolution of symptoms.

**Discussion:**

This case highlights the importance of recognizing Kounis syndrome in patients presenting with simultaneous allergic and cardiac symptoms, particularly following exposure to known triggers like contrast media. Early identification and a multidisciplinary approach are crucial for appropriate management, balancing treatment for both hypersensitivity and acute coronary syndromes.

Learning points

- Kounis syndrome is a rare clinical condition that combines hypersensitivity reaction with acute coronary syndrome, requiring early recognition and a multidisciplinary management approach.
- Acute coronary symptoms following contrast media administration should prompt suspicion of Kounis syndrome.

## Introduction

Kounis syndrome is defined as the occurrence of acute coronary syndrome triggered by hypersensitivity, typically an allergic reaction. It represents the intersection of hypersensitivity and cardiovascular disorders, where mast cell degranulation leads to the release of inflammatory mediators, causing coronary artery spasm (Type 1), atheromatous plaque erosion/rupture (Type 2), or in-stent thrombosis (Type 3). The interplay between inflammation and coronary artery pathology underscores the complex nature of this syndrome.^1,2^ Common triggers include medications (e.g. antibiotics, NSAIDs), insect bites, foods, and contrast media.^3^ It presents as a dual clinical picture of hypersensitivity (e.g. urticaria, angioedema, hypotension) and cardiac symptoms (e.g. chest pain, dyspnoea). The temporal relationship between the allergic reaction and cardiac symptoms is a key diagnostic clue. Diagnosis requires a high level of clinical suspicion, supported by electrocardiogram (ECG), troponin elevation, echocardiography (wall motion abnormalities), and coronary angiography, which assists in managing potential stenoses or in-stent thrombosis and differentiates among Kounis syndrome types. Elevated serum tryptase, eosinophil count, and IgE levels may biochemically confirm an allergy.^4,5^ This syndrome’s complexity lies in its inflammatory-cardiac interplay, necessitating rapid recognition.

## Summary figure

**Figure:**
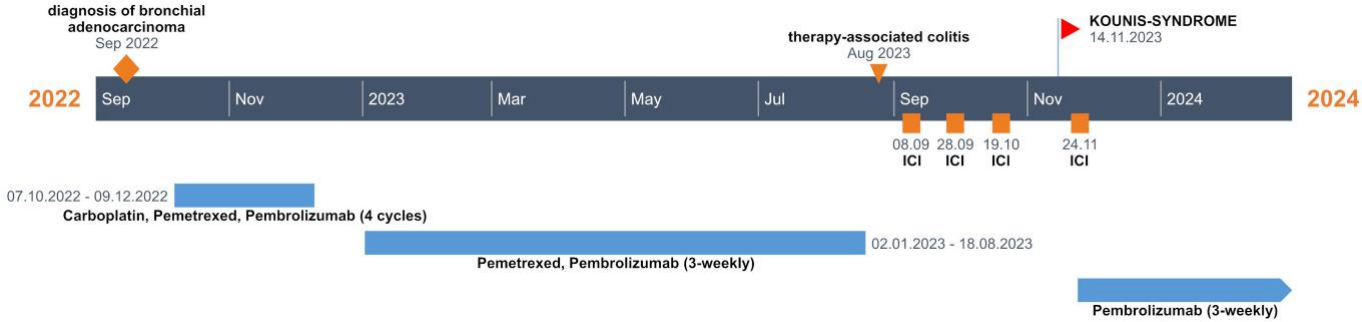


## Case presentation

A 60-year-old woman was diagnosed with bronchial adenocarcinoma in the summer of 2022. She received palliative systemic therapy with carboplatin, pemetrexed, and pembrolizumab, followed by maintenance treatment with pemetrexed and pembrolizumab, achieving sustained partial remission. Therapy-associated colitis led to a switch to pembrolizumab monotherapy, with the last dose administered 1 month before an elective computed tomography (CT) scan. She had a known acetylcysteine allergy (urticarial reaction), no prior reactions to CT contrast media, and a medical history of recurrent pneumonia, chronic obstructive pulmonary disease GOLD 1, pulmonary embolism (2022), childhood tuberculosis, and chronic smoking (10 cigarettes/day).

**Figure 1:**
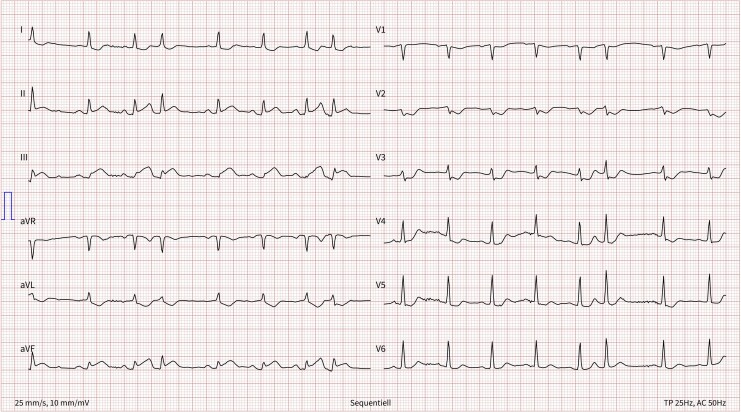
Electrocardiogram directly after onset of symptoms: ST-segment elevations in leads II, III, and AVF, as well as significant ST-segment depressions in leads V2 to V4.

She underwent a CT scan of the thorax and abdomen to evaluate the carcinoma’s progression. After receiving 85 mL of iopromide (Ultravist® 370 mg/mL) via Port-a-Cath, she developed sudden thoracic pain, cervical tightness, and orthopnoea. She exhibited tachycardia (110 b.p.m.), tachypnoea (40 breaths/min), and warm sweat. Despite receiving 6 L/min of oxygen via a mask, dyspnoea persisted, with SpO₂ at 97%–100%. Suspecting an allergic reaction, clinicians administered 500 mL of Ringer’s lactate, 2 mg of clemastine, and 125 mg of methylprednisolone intravenously. An ECG showed tachycardia, ST-segment elevations (II, III, aVF), and depressions (V2–V4), suggesting acute inferior ST-elevation myocardial infarction (STEMI) (see *Figure 1*).

**Figure 2:**
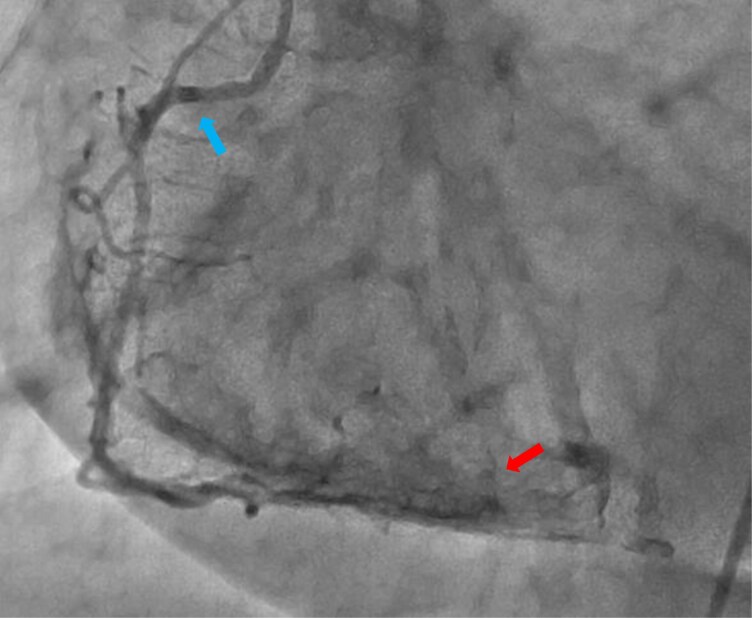
Right coronary artery: the vessel wall fully enveloped the 5F diagnostic catheter tip (indicated by the upper left arrow), causing pressure loss and myocardial contrast staining (indicated by the lower right arrow) due to injection pressure without backflow into the aorta. Intracoronary nitroglycerin administration promptly resolved these findings, confirming vasospasm.

Emergency coronary angiography revealed no significant stenosis or plaque rupture, but during catheterization of the right coronary artery, a vasospasm was observed (see *Figure 2*). It was likely provoked by catheter contact rather than the contrast medium (iodixanol 270 mg/mL, Visipaque)—otherwise, the spasm would have manifested elsewhere in the coronary artery; this resolved promptly with intracoronary nitroglycerin.

**Figure 3:**
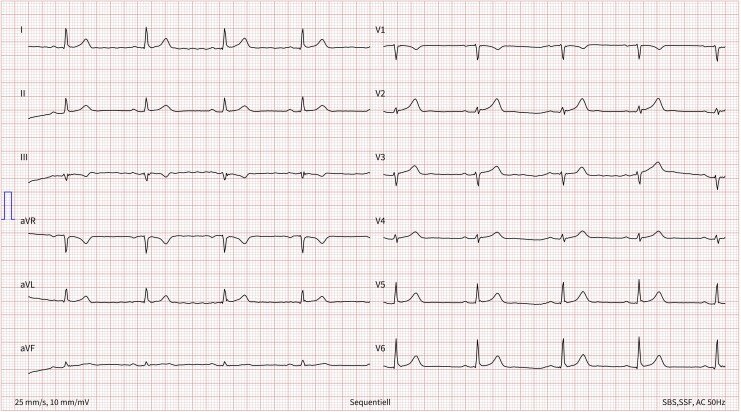
Normalized electrocardiogram 1 day after acute symptoms occurred.

Initial hs-troponin-T was 8 ng/L, peaking at 266 ng/L 4 h post-intervention and normalizing over 2 days. N-terminal prohormone of brain natriuretic peptide (NT-proBNP) rose to 1374 pg/mL, while CK and CK-MB remained normal. She was monitored in the intensive care unit overnight, with ECG changes resolving (see *Figure 3*) and no significant arrhythmias except nocturnal sinus bradycardia. The next morning, she developed severe itching and a trunk-accentuated rash, which were relieved by clemastine and methylprednisolone intravenously. Two weeks later, NT-proBNP normalized, the ECG was unremarkable, and hs-troponin-T was undetectable. Initially prescribed molsidomine was switched to diltiazem due to tachyphylaxis. Claustrophobia prevented a cardiac magnetic resonance imaging to assess immunotherapy-related cardiotoxicity. Her inpatient stay concluded uneventfully, and pembrolizumab maintenance continued without further cardiac issues.

## Discussion

The diagnosis of Kounis syndrome Type 1 was confirmed, as the administration of iopromide (370 mg/mL) during a CT scan triggered an allergic reaction with immediate vasospastic angina and a delayed cutaneous hypersensitivity reaction. Coronary angiography supported this by revealing coronary artery hyperreactivity, with a vasospasm observed during catheterization of the right coronary artery, likely induced by catheter contact rather than the contrast medium used (iodixanol 270 mg/mL), as explained in the case presentation above. The initial coronary spasm from iopromide had likely subsided before angiography—possibly due to early administration of methylprednisolone or clemastine—while the catheter provoked a new event, promptly relieved by intracoronary nitroglycerin. This sequence suggests a specific hypersensitivity to iopromide, consistent with Type 1 Kounis syndrome. Unlike iodixanol, both being iodinated contrast agents, iopromide triggered vasospasm, likely due to differences in their ligand structures rather than iodine itself. This hypothesis is supported by iodixanol’s lack of independent spasm provocation. Measuring tryptase levels^6^ could have further corroborated the allergic component.

In 2018, Raschi *et al*.^7^ published a meta-analysis summarizing drugs identified as highly likely to cause Kounis syndrome. Among the most implicated drugs, our patient had only received immunotherapy with the immune checkpoint inhibitor pembrolizumab and contrast media. Given that pembrolizumab therapy had been initiated more than a year prior, the likelihood of cardiotoxicity seems low. Studies have shown that cardiovascular events associated with immune checkpoint inhibitors typically occur within the first 90 days of therapy.^8^ Furthermore, late-onset cardiac events, if they occur, tend to manifest as heart failure or left ventricular (LV) dysfunction, rather than coronary artery spasm or myocarditis.^9^

Kounis syndrome Type 1 is a recognized cause of myocardial infarction with non-obstructive coronary arteries (MINOCA), a condition encompassing coronary, non-coronary cardiac, and non-cardiac aetiologies.^10^ Other coronary causes, such as spontaneous coronary artery dissection (SCAD), were considered, given the patient’s female sex, but excluded due to normal coronary angiography. Coronary microvascular dysfunction was unlikely due to sudden onset and to vasospasm on angiography, indicating coronary artery hyperreactivity and a vasospastic presentation. Although focal myocarditis can mimic STEMI, the combination of typical ST-elevation on ECG and vasospastic presentation with spasm on angiography makes it unlikely. Takotsubo cardiomyopathy was unlikely due to the absence of emotional or physical stress triggers, lack of LV dysfunction, and the vasospastic presentation with spasm on angiography. Pulmonary embolism was not considered the primary cause due to the vasospastic presentation with inferior ST-elevation on ECG, rapid response to nitroglycerin, and resolution of hypersensitivity symptoms with antiallergic therapy, despite a prior history in 2022. Other conditions, such as sepsis or end-stage renal failure, were excluded due to the absence of systemic illness or specific findings. This systematic evaluation confirmed Kounis syndrome as the most likely MINOCA aetiology in this case.^11^

The management of Kounis syndrome is multifaceted, addressing both the hypersensitivity reaction and the acute coronary event. There are no official guidelines for the treatment of Kounis syndrome, and therapy is based on existing guidelines for anaphylaxis^12^ and acute coronary syndrome management.^10^ However, an algorithm for Kounis syndrome management has been proposed based on a review of 288 cases.^4^ This clinically challenging scenario arises because medications used to treat one condition may exacerbate symptoms of the other,^4,5,13^ as discussed below.

The management of the hypersensitivity reaction includes the administration of glucocorticoids and antihistamines, or in the case of anaphylaxis, fluid resuscitation and epinephrine administration. Intravenous fluids needed for distributive shock from anaphylaxis can precipitate pulmonary oedema in cardiogenic shock. Epinephrine can cause coronary vasospasm and arrhythmias.^14^ In the acute setting early on, nitrates could relieve symptoms. However, close haemodynamic monitoring is crucial, as nitrates can significantly lower blood pressure, which is often aggravated by the hypersensitivity reaction. For acute chest pain relief in patients with Kounis syndrome, opioids such as morphine should be used with caution, as they can trigger significant mast cell degranulation and exacerbate the hypersensitivity reaction.^13^

In the case of our patient, no further instances of cardiac symptoms, allergic reactions, or Kounis syndrome were observed following the ongoing maintenance therapy with pembrolizumab. The CT scans for restaging were performed without contrast media; therefore, the possibility of a subsequent hypersensitivity reaction or recurrence of Kounis syndrome remains uncertain. A review of the literature revealed no clear recommendations regarding whether future CT examinations with iodinated contrast media could be safely conducted following premedication (e.g. with antihistamines and corticosteroids). Similarly, there is no evidence to suggest that switching to an alternative iodinated contrast medium would reduce the risk of recurrence, as might be the case in classic pseudoallergic reactions. This uncertainty poses a challenge for the patient’s ongoing oncological follow-up, where contrast-enhanced imaging may be clinically indicated.

## Lead author biography


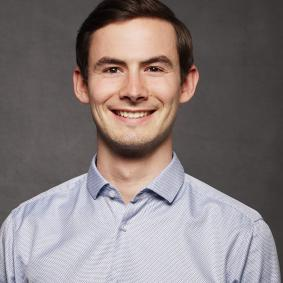


Stephan Hainzl is a doctor in training from Austria. He graduated from the Medical University of Vienna, Austria, in 2021. After beginning his career at the University Hospital in Krems an der Donau, Austria, he relocated to Switzerland, where he is pursuing his residency in Internal Medicine at St. Claraspital in Basel.

## Data Availability

The data underlying this article will be shared on reasonable request to the corresponding author, in accordance with patient privacy considerations.
